# OFD1, as a Ciliary Protein, Exhibits Neuroprotective Function in Photoreceptor Degeneration Models

**DOI:** 10.1371/journal.pone.0155860

**Published:** 2016-05-19

**Authors:** Juan Wang, Xin Chen, Fang Wang, Jieping Zhang, Peng Li, Zongyi Li, Jingying Xu, Furong Gao, Caixia Jin, Haibin Tian, Jingfa Zhang, Weiye Li, Lixia Lu, Guo-Tong Xu

**Affiliations:** 1 Department of Ophthalmology of Shanghai Tenth People’s Hospital, and Tongji Eye Institute, Tongji University School of Medicine, Shanghai, China; 2 Department of Regenerative Medicine and Stem Cell Research Center, Tongji University School of Medicine, Shanghai, China; 3 Department of Ophthalmology, Drexel University College of Medicine, Philadelphia, Pennsylvania; 4 Institute for Nutritional Sciences, Tongji University, Shanghai, China; University of Massachusetts Medical School, UNITED STATES

## Abstract

*Ofd1* is a newly identified causative gene for Retinitis pigmentosa (RP), a photoreceptor degenerative disease. This study aimed to examine Ofd1 localization in retina and further to investigate its function in photoreceptor degeneration models. Ofd1 localization in rat retina was examined using immunofluorescence. N-methyl-N-nitrosourea (MNU)-induced rats and Royal College of Surgeons (RCS) rats were used as photoreceptor degeneration models. The expression pattern of Ofd1, other ciliary associated genes and Wnt signaling pathway genes were examined in rat models. Furthermore, pEGFP-Ofd1-CDS and pSUPER-Ofd1-shRNA were constructed to overexpress and knockdown the expression level in 661W and R28 cells. MNU was also used to induce cell death. Cilia formation was observed using immunocytochemistry (ICC). Reactive oxygen species (ROS) were detected using the 2', 7'-Dichlorofluorescin diacetate (DCFH-DA) assay. Apoptosis genes expression was examined using qRT-PCR, Western blotting and fluorescence-activated cell sorting (FACS). Ofd1 localized to outer segments of rat retina photoreceptors. Ofd1 and other ciliary proteins expression levels increased from the 1^st^ and 4^th^ postnatal weeks and decreased until the 6^th^ week in the RCS rats, while their expression consistently decreased from the 1^st^ and 7^th^ day in the MNU rats. Moreover, Wnt signaling pathway proteins expression was significantly up-regulated in both rat models. Knockdown of Ofd1 expression resulted in a smaller population, shorter length of cell cilia, and lower cell viability. Ofd1 overexpression partially attenuated MNU toxic effects by reducing ROS levels and mitigating apoptosis. To the best of our knowledge, this is the first study demonstrating Ofd1 localization and its function in rat retina and in retinal degeneration rat models. Ofd1 plays a role in controlling photoreceptor cilium length and number. Importantly, it demonstrates a neuroprotective function by protecting the photoreceptor from oxidative stress and apoptosis. These data have expanded our understanding of Ofd1 function beyond cilia, and we concluded that ofd1 neuroprotection could be a potential treatment strategy in retina degeneration models.

## Introduction

Primary cilium, a microtubule-based structure protruding from the surface of most vertebrate cells, has major roles during development and in postnatal life. Sensory cilia of photoreceptors regulate the phototransduction cascade for visual processing. Cilium dysfunction is the basis for multiple human genetic disorders known as ciliopathies, which includes Joubert, Senior-Loken, Bardet-Biedl, and Oral-Facial-Digital 1 (OFD1) syndrome [1–4].

Ciliopathies are caused by mutations in genes encoding proteins required for cilia organization or function, such as *RPGR* (retinitis pigmentosa GTPase regulator) [5], *SPATA7* (spermatogenesis associated 7) [6], *POC1B* (POC1 centriolar protein B) [7], *FAM161A* (family with sequence similarity 161, member A) [8, 9], *LCA5* (Leber congenital amaurosis 5)[10], *CEP290* (centrosomal protein 290kDa) [11] and *RPGRIP1* (retinitis pigmentosa GTPase regulator interacting protein 1) [12], which are a prominent cause of severe blindness disorders due to photoreceptor degeneration.

The *OFD1* (oral-facial-digital 1) gene was initially identified in oral-facial-digital syndrome (OMIM 311200) [13] and is responsible for other ciliopathies such as Joubert syndrome [14], Simpson-Golabi-Behmel syndrome type 2 [15], and retinitis pigmentosa (RP) [16]. Importantly, most of OFD1-deficient diseases overlap with clinical spectrums that present retina dysfunction. Recently there has been an interesting report that OFD1 insufficiency causes RP in which only retina tissue suffers: deep intronic mutation, IVS9+706A>G (p.N313fs.X330) in *OFD1* is responsible for RP [16].

As a cilia protein, OFD1 localizes to both the centrosome and primary cilium [17], and OFD1, as well as CEP290, PCM-1 (pericentriolar material 1) and BBS4 (Bardet-Biedl syndrome 4) are primarily components of centriolar satellites, the particles surrounding centrosomes and basal bodies [2]. OFD1 is required for primary cilia formation, and a deletion in Ofd1 results in a loss of primary cilia [18]. in addition, Ofd1 plays a crucial role in forebrain development and in the control of dorso-ventral patterning and early corticogenesis during mouse embryonic development [19]. Thus far, there has been no any report on OFD1 function in the retina.

Recently, the Wnt signaling pathway was discovered to play important roles in retina development and disease progression, such as retinal field establishment, maintenance of retinal stem cell progenitors, retinal specification in the developing retina and homeostasis in mature retina [20–23].

Some studies have suggested that the primary cilium has a role in restraining Wnt/β-catenin signaling [24, 25]. In embryonic stem cell studies, Ofd1 mutant mouse embryonic bodies display exaggerated β-catenin-dependent pathway activation [26]. In mouse embryos, disruption of ciliogenesis via Ofd1 could up-regulate Wnt responsiveness, which suggests that primary cilium adjust to Wnt signaling transduction [27].

In the present study, we firstly examined Ofd1 localization in rat retina. Subsequently, we examined its expression in two types of retinal degeneration rat models (chemically induced and in a genetic model). The Ofd1 time course expression level with degeneration progression was investigated. Ofd1, combined with ciliary associated and Wnt signaling pathway genes were involved in both retinal degeneration rat models. Our data showed that with the exception of the role of Ofd1 in both regulating photoreceptor cilium length and number, a neuroprotective effect on the photoreceptor against oxidative stress and apoptosis was also observed.

## Materials and Methods

### Experimental Animals

The Royal College of Surgeons (RCS) rat is the first known animal with inherited retinal degeneration and widely used as an animal model of photoreceptor degeneration [28, 29]. RCS rats with *Mertk* (MER proto-oncogene, tyrosine kinase) gene deficiency results in progressive retina degeneration [30, 31], starting at 3 weeks of age until 12 weeks to totally disappeared retina cells [32]. One-week-old to 6-week-old RCS rats and age-matched SD rats (Slaccas, China) were used in this study.

To model chemical induced retina degeneration, 5-week-old SD rats were treated with intraperitoneal injection of MNU (50mg/kg), and an equal number of age-matched SD rats were injected with same volume of 0.9% saline as the control group. MNU is an alkylating agent that specifically targets photoreceptor cells causing apoptosis and cell loss within approximately 24 h after treatment [33–37]. It was acute photoreceptor apoptosis rat model.

All animals were maintained in a 12-hour alternating light-dark cycle, and food and water were provided ad libitum. MNU-treated and control groups were sacrificed post-injection on the 1^st^, 3^rd^ and 7^th^ days.

### Ethics statement

All protocols and procedures involving animals were approved by the Committee on the Ethics of Animal Experiments of Tongji University (Permit Number: TJmed-010-32). All surgical procedures were performed under anesthesia introduced by intraperitoneal injection with Pentobarbital (40 mg/kg body weight).

### Electroretinogram (ERG) Examination

To confirm that the retina degeneration model was established successfully after MNU injection, b-wave amplitude by electroretinogram recording was measured on post-injection day 1, 3, and 7 using an AVES-2000 electrophysiological apparatus (Kanghuaruiming S&T, China). The rats were placed in a dark room overnight for dark adaption before ERG test. Rats were given intraperitoneal injection with 2% pentobarbital sodium (40mg/kg), plus intramuscular injection of Sumianxin (0.5ml/kg), for general anesthesia, Tropicamide/Phenylephrine eye drop for pupil dilation and Tetracaine eye drop for local anaesthesia. Modest conductive paste was coated on the cornea of the rats. Ground electrode was implanted into the subcutaneous part of the tail root. Positive electrode was placed in the subcutaneous position between the two ears, while negative electrodes were contacted on the surface of corneas. The two eyes were stimulated twice for 0.06325 (cd*s/m) bright flash intensity simultaneously, which allowed the response of the photoreceptors to be recorded.

### Plasmid Construction

Rat and mouse *Ofd1* (NM_001106961.1, NM_177429.3) mRNA sequences were obtained from NCBI Genbank. 3 pairs of Ofd1-shRNA oligos were designed by using BLOCK-iT™ RNAi Designer, Life Technology (http://rnaidesigner.invitrogen.com/rnaiexpress), annealed, and inserted into the pSUPER-EGFP1 vector. The oligo sequences are shown in S1 Table. Scramble shRNA was inserted into pSUPER vector (without EGFP) as control.

The *Ofd1* coding sequence was amplified by PrimeSTAR HS DNA Polymerase (Takara), and cloned into the pEGFP-N2 Vector, using the following PCR primers: Forward (EcoRI): GAATTCatgaggatggctcagtccaa Reverse (BamHI): GGATCCccacatgtcatctggttctt. After sequence confirmation, the recombinant *Ofd1* construct was used for subsequent transfection experiments.

### Cell Lines

The R28 cell line consisted of rat retinal progenitor neuronal cells [38]. The 661W cell line was generated from retinal tumors of a transgenic mouse line and presented characterizations of cone photoreceptor cells [39]. Both cell lines were cultured in DMEM Low Glucose (Hyclone). Human Retinal Pigment Epithelial immortalized cells (ARPE-19 cell line) were cultured in DMEM/F12 (Hyclone) [40]. Immortalized Retinal Müller Cell Line (rMC-1) was cultured in DMEM High Glucose (Hyclone) [41]. All cell media contain 10% fetal bovine serum (Hyclone).

Ofd1-shRNA plasmids and pEGFP-Ofd1-CDS plasmids were transfected by using Lipofectamine 2000 according to the manufacturer’s instructions (Invitrogen) into R28 and 661W cell lines, respectively. MNU was added to the medium after 36 h at a concentration of 500 ug/ml and incubated for an additional 12 h [39]. The MTT assay was used to detect cell viability and proliferation.

### Quantitative Real-time PCR

The 5 x 10^5^ cells were seeded in each well of 24-well culture plate. Cells were collected with TRIzol (Takara) 48 h post-transfection. The neuroretina of RCS rat, MNU-treated rat, and control rats were also collected with TRIzol. For detail, under a dissection microscope, the anterior segments of the eye were removed. Then the eye cup was radially cut into 3 to 4 pieces from periphery to the optic nerve head. Each piece was carefully dissected and neuroretina was isolated [42].

Total RNA was extracted and reverse transcription was performed using Reverse Transcriptase M-MLV RNase H^-^ (Takara). Real-time PCR was performed in a Bio-Rad CFX Connect Real-time System by using RealMasterMix (SYBR Green) (Tiangen Biotech, China). PCR amplification was performed in duplicate (program: denaturation at 95°C for 15 min, followed by 40 cycles of 95°C for 10 s, 60°C for 40 s). All primer sequences are listed in S2 Table.

### Western Blotting Analysis

Protein expression level was analyzed using Western blotting analysis. Total proteins from cells of rat retina were extracted using RIPA plus 1% PMSF, and the protein concentration was quantified using the Pierce BCA protein assay kit (Thermo Scientific). Proteins were fractionated on an 8% SDS-PAGE gel and transferred to a 0.45-μm PVDF membrane. Membrane blocking was performed using 5% non-fat milk in Tris buffered saline supplemented with 0.1% Tween 20 (TBST). Primary antibodies were diluted in the same buffer and membrane was incubated in diluted primary antibodies overnight at 4°C, while membrane washing steps were performed with TBST. The secondary antibody was diluted in TBST and membrane was incubated at room temperature for 1 hour, and several washing steps were also subsequently performed with TBST.

The following antibodies were used in this study: β-Actin-HRP (1:5000; Proteintech), OFD1 antibody (1:200; Santa Cruz), LCA5 antibody (1:1000; Sigma Aldrich), Follistatin antibody (1:1000; Abcam), Bax antibody (1:1000; Proteintech), Bcl-2 antibody (1:1000; Proteintech) and Caspase 3 antibody (1:1000; Proteintech).

### Immunofluorescent Staining and Imaging

Cells grown on coverslips were fixed with methanol at 4°C for 10 minutes and blocked with antibody blocking buffer (0.1% Triton X-100 and 3% horse serum). Acetylated-α-tubulin (1:1,000, Abcam) was diluted in the same buffer and incubated on the cells at 4°C overnight. After washing extensively with phosphate-buffered saline (PBS), diluted anti-mouse Cy3 (1:100; Proteintech) was incubated onto the cells for 1 h at 4°C. DNA was labeled with a 1 mg/ml solution of 4’, 6’-diamidino-2-phenylindole dihydrochloride (DAPI, Sigma). After sequentially washing with PBS, coverslips were air-dried and mounted onto glass slides using DAKO mounting medium.

Age-matched rat eyes were immersion-fixed for 4 hours using freshly prepared 4% PFA. Eyes were embedded, frozen and then sectioned at thickness of 8-μm prior to incubation for immunofluorescent staining. Ofd1 (1:50, Santa Cruz), or Tmem231 (1:200, Novus) or Rhodopsin (1:500, Chemicon) was applied to each group in a humidified chamber overnight at 4°C. When dealing with fluorescein labeled Peanut Agglutinin (PNA-FITC, Sigma-Aldrich), it was applied to each section for 1h at room temperature. After washing extensively with PBS, anti-goat Cy3 (1:100; Proteintech) was applied to each section for 1h at room temperature. The DNA was labeled with a 1 mg/ml solution of DAPI. After sequentially washing with PBS, the glass slides were air-dried and mounted using DAKO mounting medium. The cells and sections were visualized using Nikon confocal Scope, which was equipped with a 60× lens.

### Measurement of cilia length and ciliated cell counts

After the cells were stained with cilium marker Acetylated-α-tubulin, the cilia length was measured under 600× magnification. Ocular micrometer (which is a round slide, in the middle of slide, a 5 mm length is equally divided into 50 parts) was used to perform the measurement. First, the ocular micrometer is corrected with a stage micrometer, such that the cilia length could then be measured with the corrected ocular micrometer. The unit of cilia length is μm.

All of the cell nuclei and its cilia within three 600× visual field were quantified. The percentage of ciliated cell number was expressed as the average percentage of ciliated cells per visual field.

### Free Radical-Scavenging Capacity Assay

Free radical production was induced by MNU in 661W cells. The cells were seeded at a density of 2000 cells/well into a 96-well plate, and incubated in a humidified atmosphere of 95% air and 5% CO_2_ at 37°C for 24 h. Next, pEGFP-Ofd1-CDS was transfected into the 661W cell line using Lipofectamine 2000 according to the manufacturer’s instructions (Invitrogen). After 48 h, the cells were washed with DMEM medium and probed with DCFH-DA (Beyotime, China) at 37°C for 20 mins. Next, MNU at 500 ug/ml was added into each well for 10 mins [39] or Rosup 1:1000 dilution was used to treat cell for 10 mins as positive control. The intensity of the fluorescence was measured using a fluorescence microscope (Leica, with monochromatic CCD) and fluorometer (Bio-Rad) at the excitation/emission wavelengths of 488/525 nm.

### Cells Apoptosis Analysis

Cells were seeded at a density of 0.5–2×10^5^ cells/well into a 24-well plate and incubated in a humidified atmosphere of 95% air and 5% CO2 at 37°C for 24 h. Next, pEGFP-Ofd1-CDS was transfected into a 661W cell line by using Lipofectamine 2000 according to the manufacturer’s instructions (Invitrogen). After 36 h, MNU of 500 ug/ml was added to each well and incubated for 12 h. For *Bax*, *Caspases3* and *Bcl-2* expression, mRNA level was determined using qRT-PCR and protein level was determined by Western blotting analysis. Primers for these genes were in S2 Table.

Moreover, the Annexin V-FITC Apoptosis Detection Kit (Sigma-Aldrich) was used to quantify apoptosis. Following over-expression of Ofd1 and treatment with MNU 500 ug/ml for 12 h, the cells were washed twice with PBS, trypsinized and resuspended in 1×Binding Buffer at a concentration of 1×10^6^ cells/ml. Subsequently, 5 μl of annexin-V-PE (Phycoerythrin) and 10 μl of PI were added to 500 μl of the cell suspension, and the mixture was incubated for 10 mins in the dark. The analyses were performed using a flow cytometer (Bio-Rad).

### Anti-oxidative stress elements detection

The neuroretina tissue of rat was isolated and lysed according to the manufacturer’s instructions. The activities of antioxidant enzymes scavenging ROS were detected in this study. Glutathione (GSH) was detected using the GSH and GSSG (Glutathione, Oxidized) Assay kits (Beyotime, China). The enzyme activities of catalase (CAT) and superoxide dismutase (SOD) were detected independently using the Catalase Assay kit (Beyotime, China) and Total Superoxide Dismutase Assay kit with WST-8 (Beyotime, China), according to the manufacturer’s instructions.

### Statistics

Data were presented as the mean ± SEMs as indicated. The data were analyzed using SigmaPlot 12.0 software. A P-value of less than 0.05 was considered statistically significant.

## Results

### Ofd1 Localization in Rat Retina

**Fig 1:**
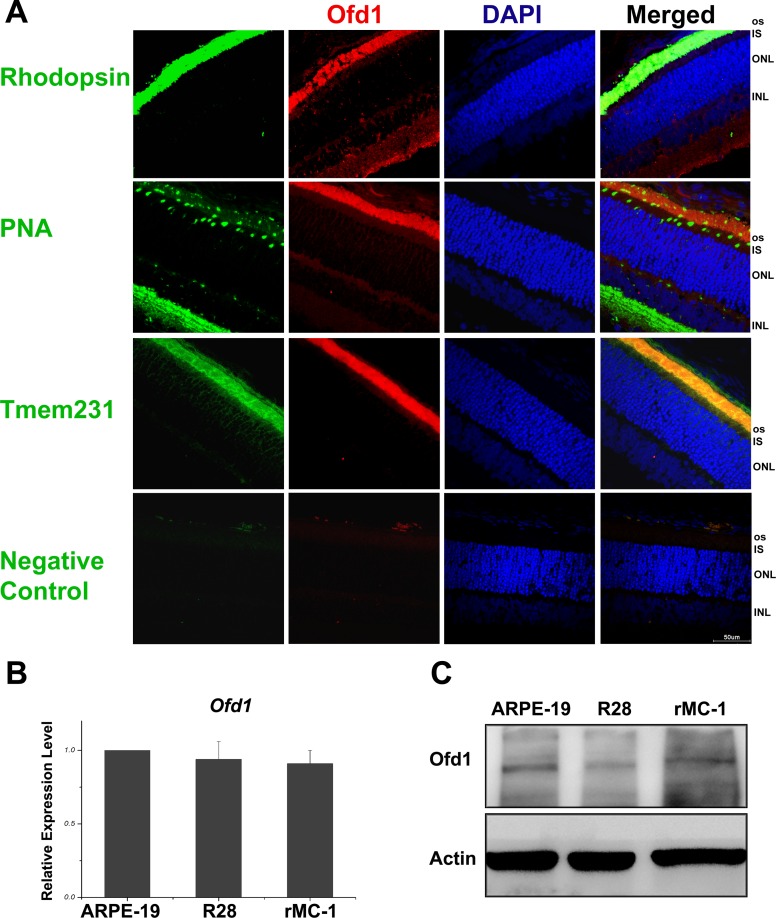
Ofd1 localization in rat retina and expression in retinal cell lines. (A) Representative confocal images of endogenous OFD1 with rod photoreceptor marker Rhodopsin, cone photoreceptor marker PNA (Peanut Agglutinin), and cilium transition zone marker Tmem231, respectively. Ofd1 immunostaining partially overlapped with photoreceptor outer segments of the retina in 2-week postnatal SD rat. Negative control: immunostaining without primary antibody incubation. Magnification 600× under confocal microscopy. Scale bar: 50μm. (B) Ofd1 mRNA expression level in retina cell lines: ARPE-19, R28 and rMC-1detected by qRT-PCR. (C) Ofd1 protein expression level rMC-1 examined using Western blotting analysis. These results confirmed that Ofd1 was expressed in these cell lines. ARPE-19: human retinal pigment epithelial immortalized cell. R28: rat retinal progenitor neuronal cell. rMC-1: immortalized retinal Müller cell. For quantification results, three replicates were performed but only one band for three representative samples per group was shown.

To examine Ofd1 localization in rat retina, we used 2-week SD rat retina frozen sections and co-stained Ofd1 with Rhodopsin which is rod photoreceptor-specific protein in retina and PNA which labeled the cone photoreceptor outer segment, inner segment and pedicles [39]. We found that Ofd1 showed disperse localization in the neuroretina, and mostly in photoreceptor outer segment. Ofd1 colocalized to Rhodopsin, and partially overlapped with PNA in photoreceptor cone inner and outer segments (Fig 1A). Besides, cilium transition zone marker Tmem231 was used to co-stain with Ofd1 and Ofd1 presented colocalized to Tmem231. Next, we examined Ofd1 expression in different retina-origin cell lines which included ARPE-19, R28, and rMC-1 cell lines using RT-PCR and Western blotting analysis. These results confirmed that Ofd1 was expressed in these cell lines (Fig 1B and 1C). However, in this study, we focused on photoreceptor cells, which are closely related with retina degeneration.

### Ofd1 and Related Ciliary Associated Genes Expression in Retinal Degeneration Animal Models

**Fig 2:**
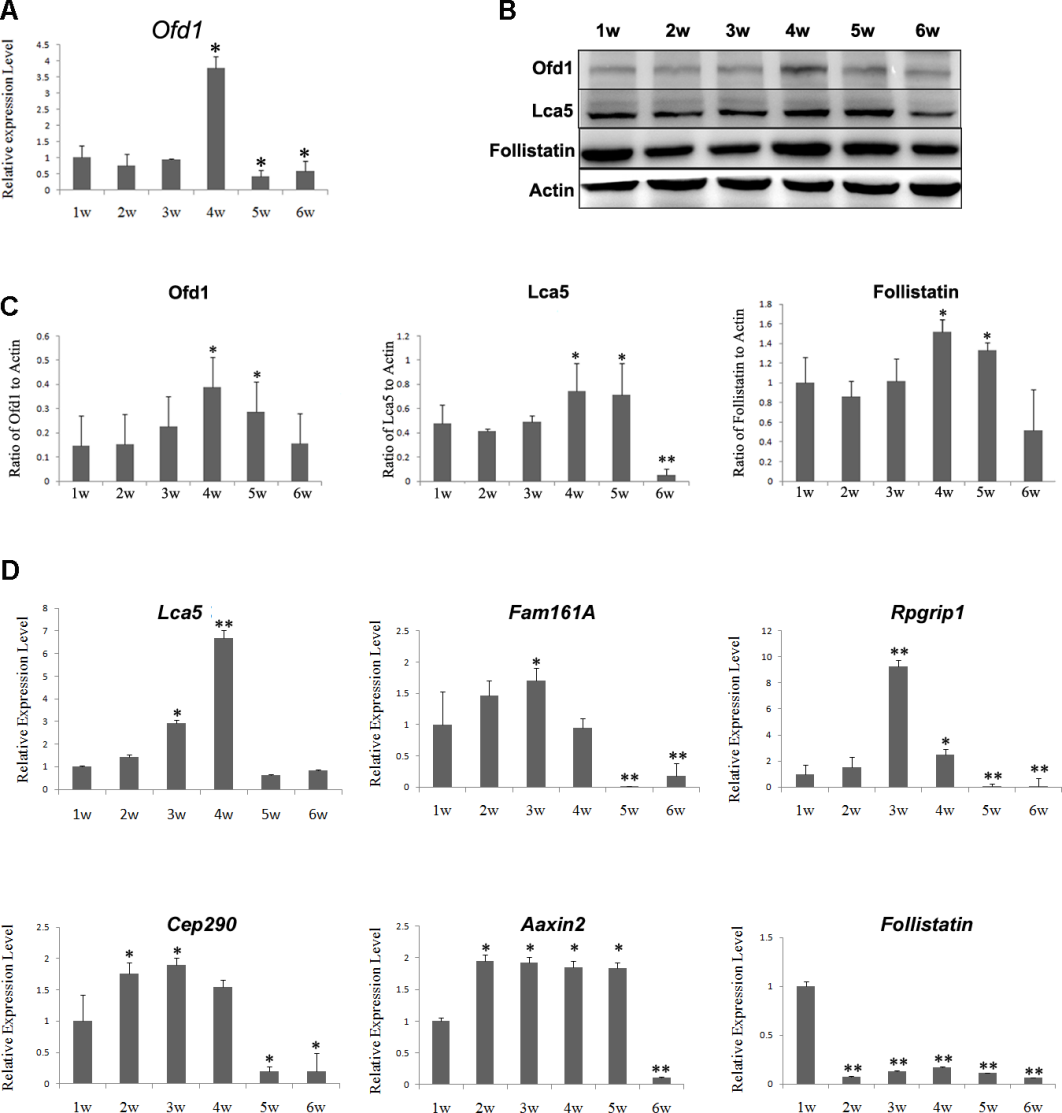
Ofd1, ciliary associated genes and Wnt signaling pathway genes expression in RCS rat retina. RCS rats were sacrificed between 1 and 6 postnatal weeks. One-week-old (1w) RCS rats were used as the control. (A) *Ofd1* mRNA expression level increased between the 1st and 4th weeks, and decreased after the 4th week, as determined by qRT-PCR. N = 4. (B) Expression level of Ofd1, Lca5 and Follistatin in RCS rat retina determined using Western blotting analysis. (C) Quantified results of Ofd1, Lca5 and Follistatin protein levels in RCS rats by QuantityOne software. Ofd1 showed the same expression pattern at the mRNA level. Lca5 and Follistatin expression presented the same pattern as Ofd1. (D) Expression level as determined by qRT-PCR of ciliary associated genes *Lca5*, *Fam161a*, *Rpgrip1*, *Cep290* and Wnt signaling pathway genes: *Axin2* and *Follistatin*. N = 4. *P<0.05, **P<0.01. For quantification results, three replicates were performed but only one band for three representative samples per group was shown.

To detect Ofd1 involvement in retinal degeneration progression, MNU-induced rat and RCS rat were used in this study in two different types of animal models. The RCS rat is genetically deficient and causes progressive retina degeneration. Our results showed that Ofd1 expression increased between the 1^st^ and 4^th^ weeks in the RCS rat retina, and decreased after the 4^th^ week, both at the mRNA and protein levels (Fig 2A and 2B).

**Fig 3:**
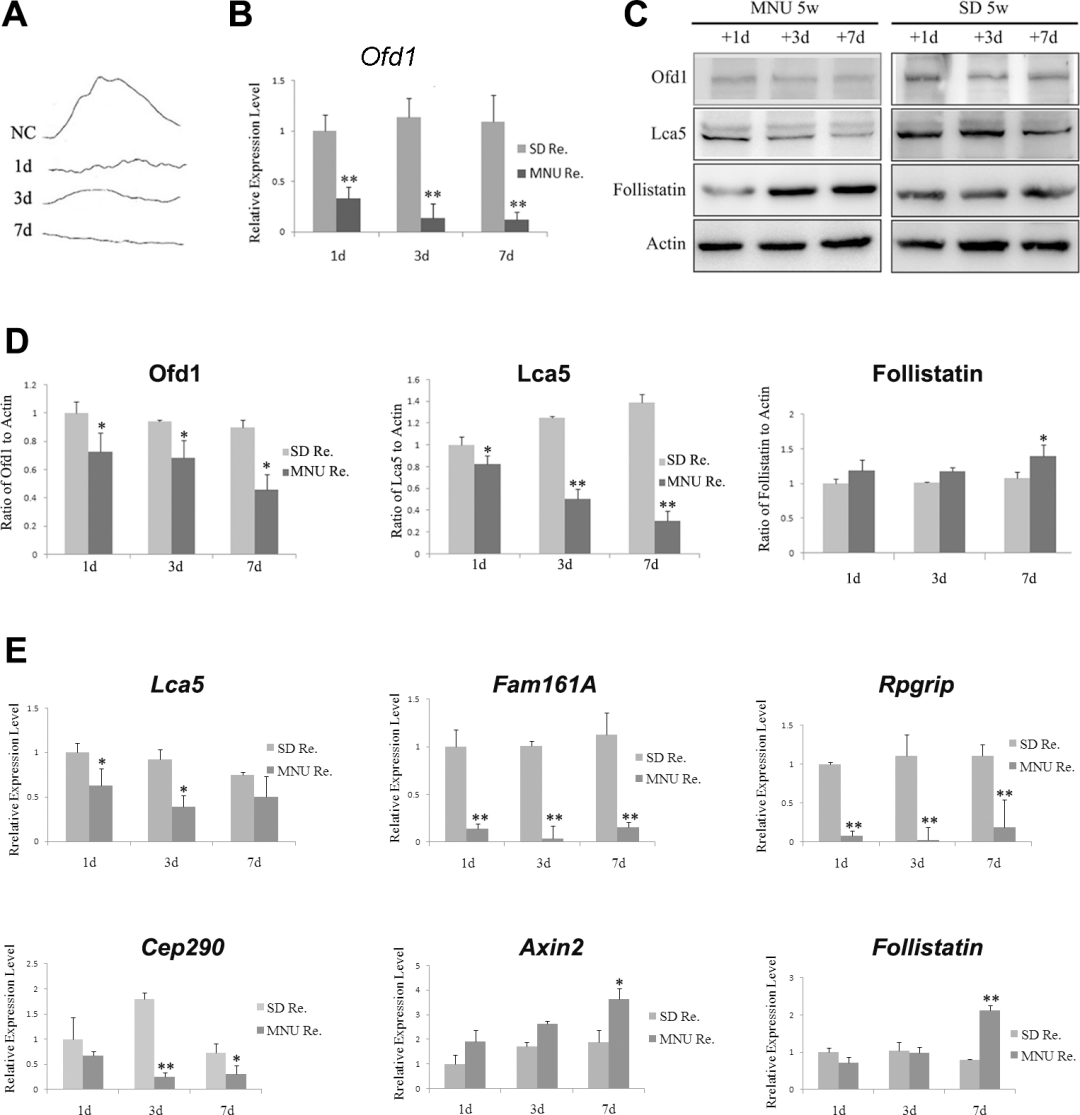
Ofd1, ciliary associated genes and Wnt signaling pathway genes expression in MNU induced retinal degeneration rat model retina. Five-week-old SD rats were injected with 50mg/kg body weight of MNU and saline, and then sacrificed on the 1 day, 3 days and 7 days post injection stages (N = 6). (A) MNU rat model was established as the ERG amplitude decreased after injection on day 1, day 3 and became flat after 7days, compared to normal SD rats. Single stimulus intense: 0.06325 (cd×s/m2); stimulus frequency: 0.5Hz. (B) Ofd1 mRNA expression in the MNU rat model was detected using qRT-PCR. Ofd1 expression decreased since the 1st day after injection and continuously decreased with time until the 7th day. (C) Protein level of Ofd1, Lca5 and Follistatin was determined using Western blotting analysis. (D) Quantified results of the protein levels of Ofd1, Lca5 and Follistatin in the MNU rat model using QuantityOne software. (E) mRNA level of ciliary associated genes and Wnt signaling pathway genes was determined using qRT-PCR. N = 6. *P<0.05, **P<0.01. For quantification results, three replicates were performed but only one band for three representative samples per group was shown.

Another model is the chemically induced acute photoreceptor apoptosis rat model. Electroretinogram was performed in this model after 1 day, 3 days and 7 days of treatment. The b-wave amplitude decreased at 1^st^ day post-injection stage and faded on the 7^th^ day indicating that MNU caused a loss in photoreceptor function and the success of model establishment (Fig 3A). In MNU-treated rat retina, Ofd1 expression decreased since the 1^st^ day after injection and continuously decreased with time until the 7^th^ day (Fig 3B and 3C).

In addition to Ofd1, other ciliary associated genes, *Lca5*, *Fam161a*, *Cep290*, and *Rpgrip1* expression were also detected. These genes were reported to interact with *Ofd1* [8, 14, 27, 39]. In RCS rats, the expression of these genes presented the same pattern as Ofd1: increased from the 1^st^ to 4^th^ postnatal week, and decreased after the 4^th^ postnatal week in general (Fig 2B and 2C). In the MNU-induced model, the mRNA and protein level (Fig 3B, 3C, 3D and 3E) of ciliary associated genes expression decreased on the 1^st^, 3^nd^ and 7^th^ day time point compared to SD normal rats.

This result suggested that Ofd1 together with Lca5, Fam161a, Cep290, and Rpgrip1 in the photoreceptor cilium complex, showed time-course-dependent changes in retinal degeneration progression in the rat models.

### Wnt Signaling Pathway Gene Expression in Retinal Degeneration Animal Models

The Wnt signaling pathway plays important roles in retina development [21, 22] and is usually activated by retinal injury [43]. Dose insufficient of Ofd1 could up-regulate β-catenin-dependent Wnt pathway activation [18, 27]. Axin2 presumably plays an important role in the regulation of the stability of β-catenin in the Wnt signaling pathway. Wnt/β-catenin positively controls follistatin expression [44].

In RCS rats, the Wnt signaling pathway gene *Axin2* was up-regulated from the 2^nd^ week, and showed a nearly 2-fold higher level of expression compared to the 1^st^ week. Axin2 presented constant high expression until the 5^th^ week, and dramatically decreased its expression in the 6^th^ week. *Follistatin* expression at the mRNA level was consistent with that of *Ofd1* expression, but at the protein level, it was strongly decreased since the 2^nd^ week (Fig 2B, 2C and 2D).

In the MNU-induced model, the Wnt signaling pathway genes, *Follistatin* and *Axin2*, increased with time (1^st^, 3^nd^ and 7^th^ day time point) and was higher compared to control SD rats at each time point (Fig 3C, 3D and 3E).

This result suggested that in the genetic and chemically induced retinal degeneration animal models, the Wnt signaling pathway was associated with degeneration progression [45].

### Ofd1 Expression Level Affected Cilia Number and Length in R28 Cells

**Fig 4:**
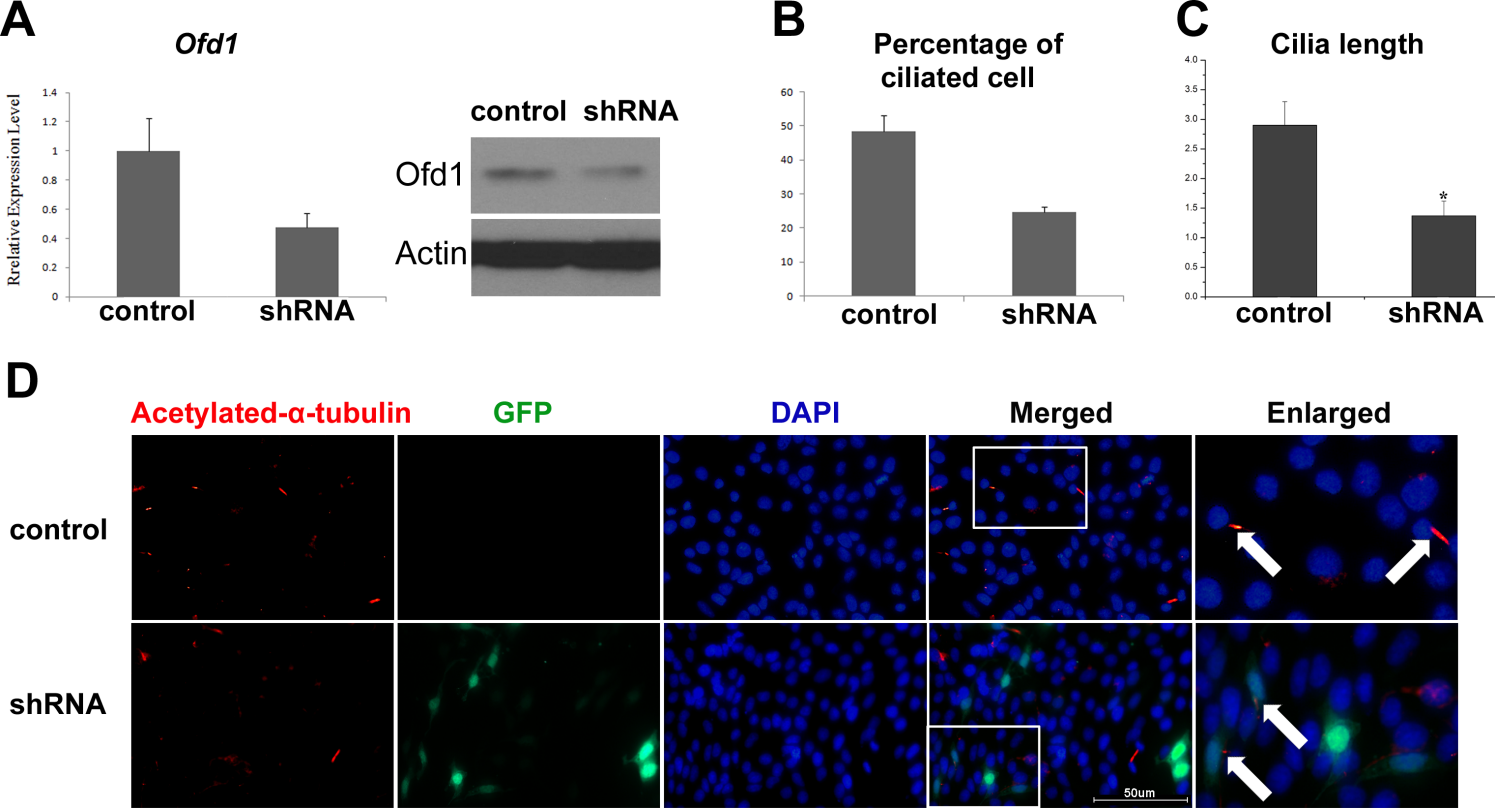
Ofd1 expression levels in cells affect cilia number and length. (A) shRNA-mediated (pSUPER-EGFP1-shRNA-Ofd1) down-regulation of Ofd1 in R28 cells determined using qRT-PCR and Western blotting analysis. Knockdown efficiency was 52%. *P<0.05. (B–C) Ciliated cell number and cilia length decreased after shRNA mediated down-regulation of Ofd1 in R28 cells (shRNA), compared with pSUPER-scramble shRNA (without EGFP) treated cells (control). *P<0.05. Unit: um. For quantification results, three replicates were performed but only one band for three representative samples per group was shown. (D) Representative image of Acetylated-α-tubulin immunostaining results. Acetylated-α-tubulin-cy3, marker of cilia axoneme, was used to demonstrate cilia in R28 cells. Cells were transfected with pSUPER-EGFP1-shRNA-Ofd1 for 48 h (shRNA), or pSUPER-scramble shRNA construct (without EGFP) as control. Magnification 600× under microscopy. Scale bar: 50μm. Enlarged: the magnified image of representative part in white box. White arrow: Acetylated-α-tubulin-cy3 staining presented cilia.

To determine Ofd1involvement in ciliary length regulation, retinal progenitor neuronal R28 cell were investigated. Ofd1 expression was up and down regulated in R28 cell respectively. We selected pSUPER-EGFP-shRNA-Ofd1 plasmid with the highest knockdown efficiency in subsequent experiments (data not shown). After Ofd1 expression was decreased in R28 cells (52% knockdown efficiency, Fig 4A), the cilia number was also decreased and cilia length was shorter as shown using Acetylated-α-tubulin immunocytochemistry (ICC) staining, which is a marker of the cilia axoneme (Fig 4D). Control was set up using pSUPER-scramble shRNA (without EGFP) transfected R28 cells. To quantify this finding, the ciliated cell percentage decreased to approximately 50%, compared to control R28 cell (Fig 4B). The cilia length shortened to approximately 50% compared to control cells (Fig 4C). These findings indicated that the Ofd1 expression level caused direct cilia malformation, which might result in defects in photoreceptor cell.

### Ofd1 Protection on Photoreceptor from Oxidative Stress via Decreasing ROS Production

**Fig 5:**
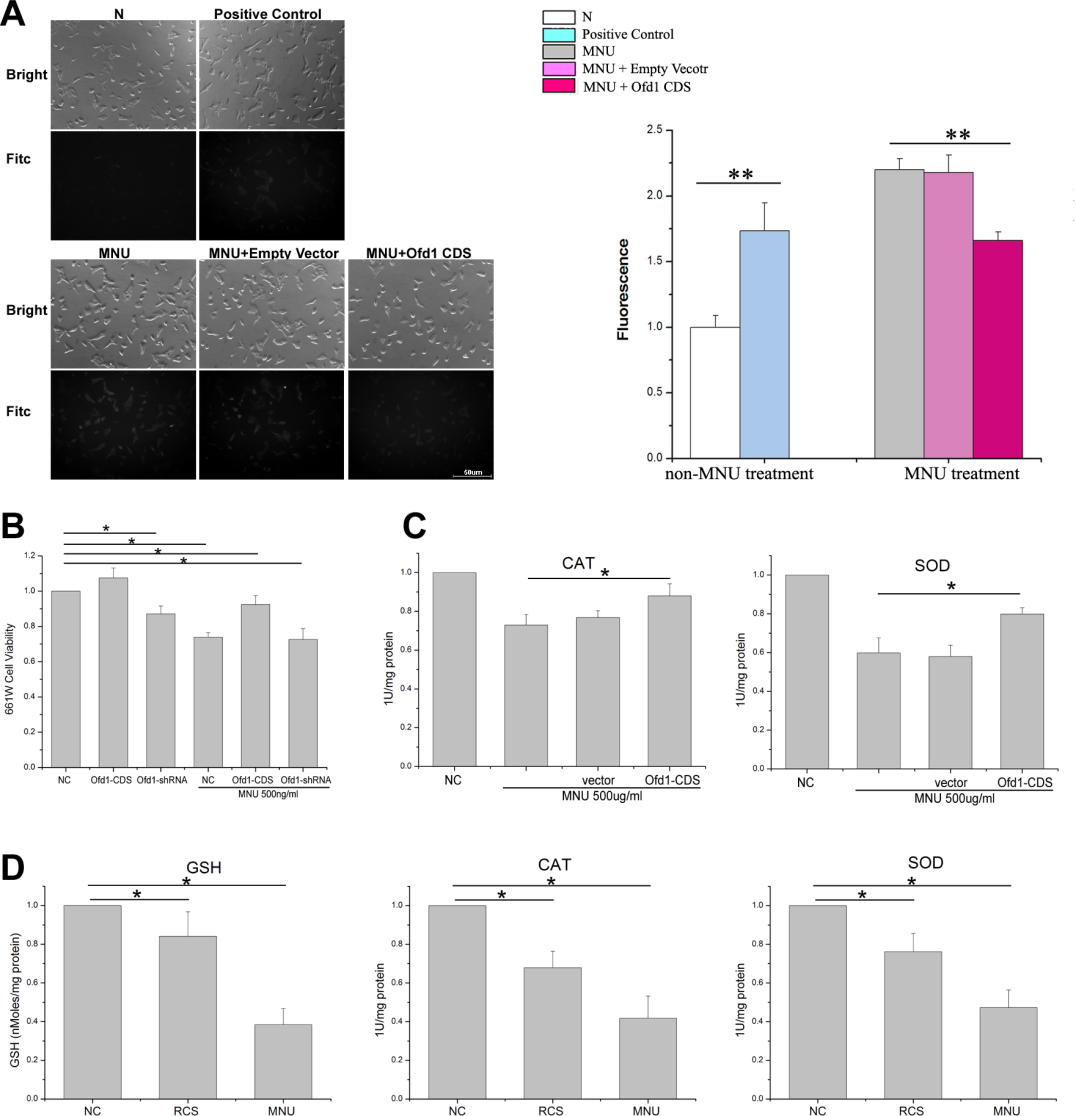
Ofd1 protection of photoreceptors from oxidative stress via decreasing ROS production. (A) The intracellular oxidant DCFH of MNU-induced ROS in 661W cells observed by green fluorescence using Leica fluorescence microscope (with monochromatic CCD), and measured using fluorometer at wavelength of 488/525 nm. ROS production was stimulated 10 mins by 500 ug/ml MNU or ROSup as positive control. N: non-treated cells. MNU: MNU 500 ug/ml treated 661W for 10 mins. MNU+Empty Vector: after 661W transfected with pEGFP vector for 48h, MNU 500 ug/ml treated for 10 mins. MNU+Ofd1 CDS: after 661W transfected with pEGFP-Ofd1-CDS for 48h, MNU 500 ug/ml treated for 10 mins. Scale bar: 50μm. (B) Cell viability and proliferation detection in 661W cell using the MTT assay. Ofd1 expression was up- or down-regulated by plasmid transfection by Lipofectamine 2000. Ofd1-CDS was used to overexpress Ofd1, and Ofd1-shRNA was used to inhibit expression. After transfection for 36 h, 500 ug/ml MNU was added and incubated for additional 12 h. NC: normal control. * p<0.05. n = 6. (C) These results showed that the SOD and CAT activities decreased by 40% and 30% in MNU-treated cells (MNU), but was partially attenuated by Ofd1 overexpression (Ofd1-CDS+MNU). The difference between the MNU group and Ofd1-CDS+MNU group was significant. *: p < 0.05. n = 4. (D) Antioxidant enzymes amount or activity detection in RCS rat at 6 weeks (RCS) compared to age-matched normal SD rat as the control (NC), MNU-induced rat at 7 days (MNU) compared to samples obtained from age-matched no- treated rats as the control (NC). The two NC are the same, 6-week normal SD rat. * p<0.05. n = 4.

To examine the potential mechanism of Ofd1 involvement in retinal degeneration, the 661W cell line with characterizations of cone photoreceptors was used. To mimic MNU-induced photoreceptor cell death in vitro, MNU was directly added to the 661W cell medium, and we found that MNU 500 ug/ml treatment caused a toxic effect in 661W cells using the MTT assay (Fig 5B). Knockdown of Ofd1 expression in 661W cells caused lower cell viability, while overexpression of Ofd1 had no effect. However, after MNU treatment, overexpression of Ofd1 could partly attenuate the MNU toxic effect in 661W cells.

We hypothesized that photoreceptors were protected by Ofd1 from oxidative stress via decreasing ROS production. First, we detected the ROS levels in MNU-treated 661W cells using the DCFH-DA probing method. Intracellular DCFH (non-fluorescent) was oxidized to 2’, 7’-dichlorfluorescein (DCF, fluorescent) by intracellular ROS. Compared with 661W transfected with empty vector, the fluorescence levels were significantly decreased in 661W cells with exogenous Ofd1 over-expression (Fig 5A), which indicated overexpression of Ofd1 could decrease ROS production in 661W cells.

To determine if Ofd1 is involved in oxidative stress in 661W cell, antioxidant enzymes activities were analyzed in the cell pellet after Ofd1-CDS transfection to overexpress Ofd1 and/or MNU treatment, compared to no-treated 661W cells (NC). These results showed that the SOD and CAT activities had decreased by 40% and 30%, respectively, in MNU-treated cells (MNU), but was partially attenuated by Ofd1 overexpression (Ofd1-CDS+MNU). The difference between the MNU group and Ofd1-CDS+MNU group was significant (p < 0.05) (Fig 5C). Total GSSG was too low following Beyotime kit detection, such that the GSH content could not be calculated (data not shown). These results demonstrated that Ofd1 overexpression could increase the amount/activity of reactive oxygen scavenging enzymes (SOD, CAT), to alleviate MNU-induced ROS production in 661W cell.

Further, to determine if oxidative stress is involved in two types of retinal degeneration rat models, the GSH level and activities of CAT and SOD in neural retinas were analyzed, including the retina sample obtained from RCS rat at 6 weeks (RCS) compared to an age-matched normal SD rat as the control (NC), and MNU-induced rat at 7 days (MNU) compared to a sample obtained from an age-matched no-treated rat as control (NC). These results demonstrated a reduction in 16–33% of GSH, SOD activity and CAT activity in the RCS group, and a reduction in 53–62% of GSH, SOD and CAT activity by 40% in the MNU group (Fig 5D).

As previously reported, oxidative stress plays an important role in the pathogenesis of retinal degeneration diseases, such as diabetic retinopathy (DR) [46]. In MNU-induced photoreceptor degeneration model, MNU-induced apoptosis may result from oxidative stress [47].

### Ofd1 Protection on Photoreceptor from Decreasing Apoptosis

**Fig 6:**
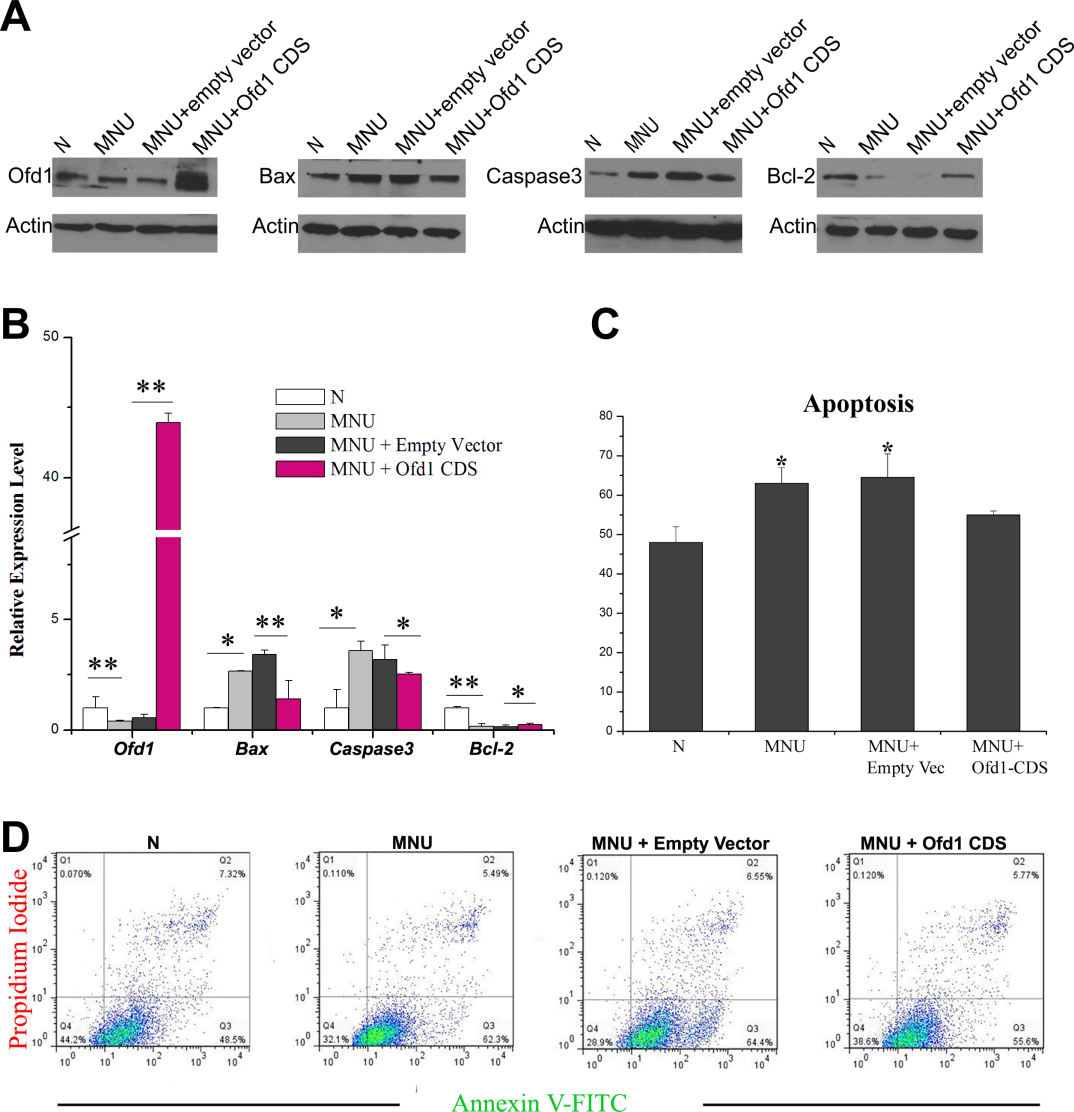
Ofd1 protection on photoreceptor via decreasing apoptosis. (A) Western blotting analysis to detect protein levels of Bax, Caspase3 and Bcl-2 in 661W cell. 661W cells with Ofd1 over-expression had lower expression of Bax (nearly half), lower Capsase3 expression (approximately 20% lower), and higher Bcl-2 expression (2-fold). (B) qRT-PCR to detect mRNA levels of apoptotic genes (*Bax*, *Caspase3* and *Bcl-2*) in 661W cell. (C-D) Cells apoptosis was analyzed flow cytometer in 661W cell. Q3: the percentage of early apoptotic cells. Q1: the percentage of late apoptotic cells. N: non-treated cells. MNU: MNU 500 ug/ml treated 661W for 10 mins. MNU+Empty Vector: after 661W transfected with pEGFP vector for 48h, MNU 500 ug/ml treated for 10 mins. MNU+Ofd1 CDS: after 661W transfected with pEGFP-Ofd1-CDS for 48h, MNU 500 ug/ml treated for 10 mins. (C) is the statistical result of (D), which shows percentage of apoptotic cells in 661W. Cell percentage undergoing apoptosis significantly increased after MNU treatment (approximately 35% increase), but returned back to normal levels after Ofd1 overexpression. For quantification results, three replicates were performed but only one band for three representative samples per group was shown.

With the exception of a decrease in ROS production, the protective effect of Ofd1 on the photoreceptor could be various and complicated. Based on MNU-specifically induced photoreceptor apoptosis and cells death [39, 48], a second potential mechanism might be Ofd1 alleviated apoptosis. Cell apoptosis was analyzed using qRT-PCR and Western blotting analysis to detect the mRNA levels and protein levels of apoptotic genes (*Bax*, *Caspase3* and *Bcl-2*) (Fig 6A and 6B). 661W cells overexpressed with Ofd1 had lower expression of Bax (nearly half), lower Capsase3 expression (approximately 20% lower), and higher Bcl-2 expression (2-fold).

To confirm this result, Annexin V-FITC was used to mark apoptotic cells and quantitative determination by flow cytometry was performed. Quantitatively, the percentage of cells undergoing apoptosis would significantly increased after MNU treatment (approximately 35% increase), but returned back to normal levels after Ofd1 overexpression (Fig 6C and 6D).

## Discussion

The present study is the first study to examine Ofd1 localization and its cilia associated function in retina tissue, and it also further investigates the potential mechanism underlying retina degeneration in animal model. We demonstrated that Ofd1 exerted its neuroprotective effects via both anti-oxidative stress and anti-apoptotic mechanisms.

As a known cilia protein [49] but no report in retina tissue, our results showed that Ofd1 was localized to the photoreceptor outer segments as shown by immunostaining in rat retina. Retina connecting cilium connects the inner and outer segments of the photoreceptor, mediating bi-directional transport of phototransducing proteins required for vision. The photoreceptor outer segments are considered to be specialized sensory cilia [50]. Our results of Ofd1 localize to photoreceptor outer segment are in accordance with Ofd1 as a cilia protein. Further, in retina cells, Ofd1 knocked down in photoreceptor progenitor R28 cells affected cilia number and length. In this case, the cilium is the primary functional site for Ofd1 and disruption of Ofd1 protein would affect the ciliary structure in retina. OFD1 is required for primary cilia formation, which has been proved by previous literatures. Tang et al. (2013) [51] demonstrated that autophagic degradation of Ofd1 at centriolar satellites promotes primary cilium biogenesis suggesting that Ofd1 negatively regulate cilia formation, but Hunkapiller J et al (2010) stated a deletion in Ofd1 results in a loss of primary cilia in embryonic stem cell, which was consistent with the finding reported here. We speculated that in different cell lines, in different stimulation, the role of Ofd1 in ciliogenesis may be different.

To mimic retina degeneration progression, genetic and chemically induced rat models were applied in this study: RCS rats and MNU-induced retinal degeneration rats. RCS rats harbors a mutant in Mertk gene, which plays an essential role in phagocytosis and ingestion of outer segments by RPE cells and subsequently results in progressive photoreceptor degeneration beginning at 21 days postnatally and deteriorates gradually [30, 31]. MNU-induced acute photoreceptor degeneration is caused by apoptosis and cell loss within approximately 24 hours after treatment [33–37]. These two retinal degeneration models are completely different, and thus it was reasonable to propose that Ofd1, other cilia-related genes, and Wnt signaling pathway genes are differentially expressed. Because the primary diseased cells in the RCS rats are RPE instead of the photoreceptor, altered expression of Ofd1 may be compensatory and secondary to an RPE defect. The altered expression level of Ofd1 with disease progression in both models indicated that Ofd1 was closely linked to retinal degeneration [5]. Notablely, Ofd1 expression in RCS rat reached the highest level at the 4^th^ week postnatal stage and then decreased at both mRNA and protein levels. This is consistent with the apoptosis peak at the 4^th^ week in RCS rats [52], which connect Ofd1 protection effect with decreasing apoptosis.

It is reported that Ofd1 interacted with other ciliary associated protein components (Lca5, Fam161a, Cep290, and Rpgrip1) [8, 14, 27, 39, 53]. Mutations in these ciliary genes are implicated in photoreceptor degeneration related ciliopathies [16, 54], such as *RPGRIP1* in cone dystrophy (CD)/cone-rod dystrophy (CRD), *LCA5* and *CEP290* in Leber congenital amaurosis (LCA), *Fam161A* and *OFD1* in RP. This finding also provided a evidence that *OFD1* mutation can be a cause of retina degeneration disease in human, although there is only one paper reported that deep intronic mutation in *OFD1* responsible for RP patient [16].

From our results Axin2 and follistatin expression, Ofd1 and β-catenin-dependent Wnt signaling pathway both involved in two retina degenerative models. Axin2 and Follistatin expression were up-regulated while Ofd1 expression was down-regulated in the MNU-induced animal model in this study. However, in the RCS rats, Follistatin expression was not consistent with the Axin2 pattern. Moreover, the Follistatin expression pattern was not consistent at the mRNA and protein levels. Thus, the association between Ofd1, Axin2 and Follistatin in RCS rats requires further investigation.

To examine the function of Ofd1 in photoreceptors beyond a single ciliary protein, we speculate that it may be closely related to oxidative stress. MNU-induced photoreceptor cell death is associated with oxidative stress and apoptotic mechanisms [39, 48, 55], which involves up-regulation of Bax and Caspase3 and down-regulation of Bcl-2. Over-expression of Ofd1 in 661W cells could reduce intracellular ROS production and the number of Annexin V-positive apoptotic cells, indicating that Ofd1 is resistant to oxidative stress and decreasing apoptosis in photoreceptors. In this study, Ofd1 treatment exhibited neuroprotective effects on the apoptosis of photoreceptor cells 661W, but its mechanism remains unclear and further investigations are needed.

For human ciliopathy, such as some photoreceptor degenerative disease, a large number of causative genes have been identified using the wide application of next generation sequencing technologies. These technologies will provide insight into the mechanism of ciliopathy. Our work has greatly expanded our understanding of the function of ciliary proteins in the specific ciliopathic context beyond its cilia structure and mechanistic analysis in retina degeneration. These findings will greatly help to develop novel therapeutic strategies for retinal degeneration.

## Supporting Information

S1 Table The oligo sequences of rat Ofd1-shRNA. (DOC)

S2 Table qRT-PCR Primers of *Ofd1*, Ciliary Associated Genes and Wnt Signaling Pathway Genes and Apoptosis Related Genes. (DOC)
