# Supplementary material for: OFD1, as a Ciliary Protein, Exhibits Neuroprotective Function in Photoreceptor Degeneration Models
Source: PLoS One. 2016 May 19;11(5):e0155860. doi: 10.1371/journal.pone.0155860 (PMC4873209; doi:10.1371/journal.pone.0155860)
Supplement: S1 Table — (DOC) [file pone.0155860.s001.doc]

**Supporting Information**

S1 Table. The oligo sequences of rat Ofd1-shRNA

|  | **Primers (5'-3')** |
| --- | --- |
| rOFD1-shRNA-BglII-1F | GATCCCCGCAAACTCAGACAGCCCTAGATTCAAGAGATCTAGGGCTGTCTGAGTTTGCTTTTTA |
| rOFD1-shRNA-HindIII-1R | AGCTTAAAAAGCAAACTCAGACAGCCCTAGATCTCTTGAATCTAGGGCTGTCTGAGTTTGCGGG |
| rOFD1-shRNA-BglII-2F | GATCCCCGCCTATTGAGTGGCAAGATGGTTCAAGAGACCATCTTGCCACTCAATAGGCTTTTTA |
| rOFD1-shRNA-HindIII-2R | AGCTTAAAAAGCCTATTGAGTGGCAAGATGGTCTCTTGAACCATCTTGCCACTCAATAGGCGGG |
| rOFD1-shRNA-BglII-3F | GATCCCCGGTTATGGCAGGTGCAGTTGTTTCAAGAGAACAACTGCACCTGCCATAACCTTTTTA |
| rOFD1-shRNA-HindIII-3R | AGCTTAAAAAGGTTATGGCAGGTGCAGTTGTTCTCTTGAAACAACTGCACCTGCCATAACCGGG |
