# Supplementary material for: OFD1, as a Ciliary Protein, Exhibits Neuroprotective Function in Photoreceptor Degeneration Models
Source: PLoS One. 2016 May 19;11(5):e0155860. doi: 10.1371/journal.pone.0155860 (PMC4873209; doi:10.1371/journal.pone.0155860)
Supplement: S2 Table — (DOC) [file pone.0155860.s002.doc]

**Supporting Information**

S2 Table. qRT-PCR Primers of OFD1, Ciliary Associated Genes and Wnt Signaling Pathway Genes and Apoptosis Related Genes

| **Gene** | **Gene ID** | **Primers(5’-3’)** |
| --- | --- | --- |
| Rat Gapdh | NM_017008.4 | GCCTCGTCTCATAGACAAGAT |
|  |  | TCCACT TTGTCACAAGAGAAG |
| Ofd1 | NM_001106961.1 | GCAGGTGCAGTTGTGTCAAG |
|  |  | CTGAAGGCCTTTTCCAAGCG |
| Lca5 | NM_001013954.1 | GGAAAGTACAGCGACGACCA |
|  |  | TCCGCCTCGTCTTGGAATTT |
| Fam161a | NM_001013876.2 | ACCCCAGAAGTCCTGCAATG |
|  |  | GAAGGATTAGGCCTGCGTGT |
| Rpgrip1 | NM_020366.3 | AACCAGGTGTCCAAGGCAAA |
|  |  | AGTGCTGAATGGGGTGATGG |
| Cep290 | NM_001135755.1 | TGTTTGGGAAGAGTTGGCGG |
|  |  | ACTCTTCTTGACGTGGCAGTT |
| Axin2 | NM_024355.1 | ATCCAGCAAAACTCTCCGGG |
|  |  | TACTCCCCATGCGGTAAGGA |
| Follistatin | NM_012561.1 | AACGAATGTGCGCTCCTCAA |
|  |  | GCAGGCACTGGAGTAAGTCA |
| Mus Gapdh | NM_001289726.1 | CCCCTTCATTGACCTCAACTACA |
|  |  | TCCCATTCTCAGCCTTGACTGT |
| Caspase3 | NM_001284409.1 | GAGCTTGGAACGGTACGCTA |
|  |  | ATGCCCATTTCAGGATAATCCATTT |
| Bax | NM_007527.3 | GCGTGGTTGCCCTCTTCTACTTTG |
|  |  | AGTCCAGTGTCCAGCCCATGATG |
| Bcl-2 | NM_009741.4 | GTCCCGCCTCTTCACCTTTCAG |
|  |  | GATTCTGGTGTTTCCCCGTTGG |
